# Hear Our Voice: A Photo‐Elicitation Study to Explore the Social Participation of Older People With Mild/Moderate Intellectual Disability

**DOI:** 10.1111/jar.70083

**Published:** 2025-06-29

**Authors:** Zuyu Wang, Andrew Sommerlad, Joan K. Monin, Angela Hassiotis, Gill Livingston

**Affiliations:** ^1^ Division of Psychiatry University College London London UK; ^2^ North London NHS Foundation Trust London UK; ^3^ Department of Social and Behavioral Sciences Yale School of Public Health New Haven Connecticut USA

## Abstract

**Background:**

People with intellectual disability are more socially isolated than the general population.

**Aims:**

To explore the social participation experiences and enablers and barriers of older people with mild/moderate intellectual disability.

**Methods:**

Following co‐production of the research with an advocacy group, we purposively recruited people aged over 50 with mild/moderate intellectual disability, using photo‐elicitation methods and qualitative interviews, analysed thematically.

**Results:**

We recruited 14 participants (5 women) from London, UK. Themes were (1) negotiating social belonging in a world of barriers, (2) obstacles go beyond intellectual disability, (3) support must be tailored and personalised. Their experiences of discrimination made them afraid to go out and often to mix with people without disabilities. However, they enjoyed socialising, appreciating variety, social connection and learning.

**Conclusion:**

People with intellectual disability wished to socialise but encountered multiple obstacles. Such barriers limit meaningful community engagement and inclusivity, underscoring the need for interventions to combat isolation.

**Summary**

- People with mild or moderate intellectual disability (also called learning disability) have highly valued and varied activities. However, we found they need support adapted to their specific needs. They often have challenges in communication, understanding and physical problems. They frequently have lived through traumatic events or stigma, which make them afraid to go out on their own and to socialise.
- Their past and present experiences of discrimination and abuse reduce the ability of people with intellectual disability to enjoy the present.
- Research that uses photographs can help people with intellectual disability talk about how they manage their social relationships and friendships.

## 1 Introduction

Social participation refers to a person's involvement in activities with others in community life and in important shared spaces (Levasseur et al. 2022) and is important for everyone, partly because it is associated with later‐life quality of life and cognition (Wang et al. 2024). However, it can be challenging for people with intellectual disability to initiate and maintain social participation. Compared to people without intellectual disability, people with intellectual disability experience significantly higher rates of loneliness, low perceived social support and social isolation, which drives poor well‐being (Emerson et al. 2021). Older adults with intellectual disability are likely to face increased exclusion and barriers to social participation (McCausland et al. 2023) due to loss of employment opportunities and age‐related health and mobility issues (Stancliffe and Hall 2023). Among people with intellectual disability, increased loneliness has been associated with higher rates of depression, mental health problems and poorer physical health (Alexandra et al. 2018).

Even when people with intellectual disability participate in community activities, they may still feel excluded and lack a sense of belonging. They may also struggle to experience reciprocity in their interpersonal relationships, both of which are essential aspects of social inclusion (Fulton et al. 2021). In addition, people with intellectual disability may have difficulty with abstract concepts, necessitating careful planning and communication strategies to ensure effective engagement (Scheffers et al. 2024). Therefore, research and practice on social participation of people with intellectual disability have extended from focusing on physical inclusion and also emphasising meaningful social inclusion (Amado et al. 2013), where people with intellectual disability feel acknowledged and valued (Meininger 2010; Merrells et al. 2018).

Therefore, there is a need to identify approaches to support the social participation of people with intellectual disability, particularly in older age when they may have less support from close family and report higher loneliness (Egan et al. 2022; Lehmann et al. 2013), to reduce social isolation and loneliness. We examined social participation in people with intellectual disability aged over 50 to gain insights into their social participation.

### 1.1 Aims

We aimed to explore the barriers and facilitators to social participation for older community‐dwelling people with intellectual disability by using photo‐elicitation interviews in order to inform future interventions to promote social participation in this population.

## 2 Methods

### 2.1 Study Design

In this study, we used the photo‐elicitation method to explore the experience of social participation in people with mild/moderate intellectual disability. Photo‐elicitation is a method which involves participants taking photographs (Samuels 2004) to visually represent their experience, using photographs as prompts could help articulate feelings and thoughts that might otherwise be challenging to express (Scheffers et al. 2024). It can be a useful tool in facilitating communication with participants with intellectual disability by forming a basis for contextualising and more in‐depth by enabling researchers to guide discussions of the context, circumstances and meaning of photographs from the perspectives of participants (Frith and Harcourt 2007).

### 2.2 Ethical Approval

This study was approved by NHS Health Research Authority: West Midlands–South Birmingham Research Ethics Committee (23/WM/0091).

### 2.3 Participants and Setting

Initially, we contacted a list of NHS settings and third sectors, then we recruited participants from those who replied to us, including two inner London National Health Service intellectual disability teams and three London‐based third‐sector providers. After that, we consulted the staff and professional carers, asking them to approach potential participants who had capacity to consent and might be interested in taking part. Eligible participants were people with mild or moderate intellectual disability aged ≥ 50 years old living with family or in supported living who were English speakers. They were from a range of ethnicities, ages and genders and had capacity to give informed consent to research. We provided all tools (e.g., cameras, vouchers for their time) to make sure no one was excluded because of lack of financial resources. During the recruitment process, the research team had meetings to discuss the background of participants to ensure purposeful recruitment for diversity. We recruited participants until reaching theoretical sufficiency, meaning their narratives started overlapping with those of previous participants (Saunders et al. 2018).

### 2.4 Procedures

We provided potential participants with easy‐read versions of participant information sheets (PIS) about the study before the initial meeting with the researcher (Z.W.). If they were interested in the study, Z.W. arranged a meeting at a place and time convenient to the participants, during which she answered questions about the study.

#### 2.4.1 Consent

If participants were still interested in taking part, the researcher met the participants again, at least 48 h after the first meeting and obtained informed consent. The researcher lent participants a digital camera and showed them and a carer known to the participant (if they were willing to) how to use it. She asked them to take around 10 photographs when they were socialising with others during the following 2 weeks. They could use their smartphones if they preferred. Participants could have support from carers, and the researcher also reminded them to use the cameras either by telephone or in person. Additionally, the researcher offered extra time (1–2 weeks) to any participants who were unable to take many photographs within the initial 2 weeks.

In this study, participants chose when to conduct their interviews and whether to do them alone or with someone they felt comfortable with, such as a carer or a friend, but all interview questions were addressed to the person with intellectual disability to maximise their engagement.

### 2.5 Reflexivity

Throughout the study, we considered how the researchers' backgrounds and experiences shaped the design, conduct and analysis of the study and influenced the depth and nature of the data collected during the interviews. All authors were experienced in qualitative research, Z.W. held a master's degree in social work and has previously carried out research with older people with disabilities in non‐UK communities. Her previous role as a community social worker informed her expectations of potential barriers of older people with cognitive impairments and her approach to the research. It also helped her identify potential participants with dementia who were not eligible for this study. Specifically, her experiences gave her insights into challenges such as social isolation, difficulty accessing resources and stigma, which she anticipated might also emerge in this study. These expectations guided her to pay particular attention to identifying and understanding similar barriers throughout her research. Drawing on her social work experience, Z.W. recognised the significant impact that a supported living home could have on the lives of people with intellectual disability and how support could make a difference.

Z.W. is a non‐native English speaker who has lived and studied for her PhD, but not worked as a social worker in the United Kingdom for 3 years. She had access to Photosymbols and was trained in accessible communication from the Camden Learning Disability Service. She realised from her work how difficult it could be for participants to contribute if they did not have strong verbal skills. Therefore, she used simple sentences and appropriate vocabulary to support understanding in the interviews. There were no language barriers occurred during the interviews. Besides, she was perceived by some participants as a friend and someone who might need their help. For example, participants said to her, ‘You are my second best friend’ (P01). These possibly fostered openness and trust during interviews.

### 2.6 Measures

We collected demographic and clinical information about participants in a structured form, including age, gender identity, marital status, self‐identified ethnicity, self‐reported mental health diagnoses, severity of intellectual disability if known and daytime occupation if applicable.

#### 2.6.1 Photo‐Elicitation Interviews

**FIGURE 1:**
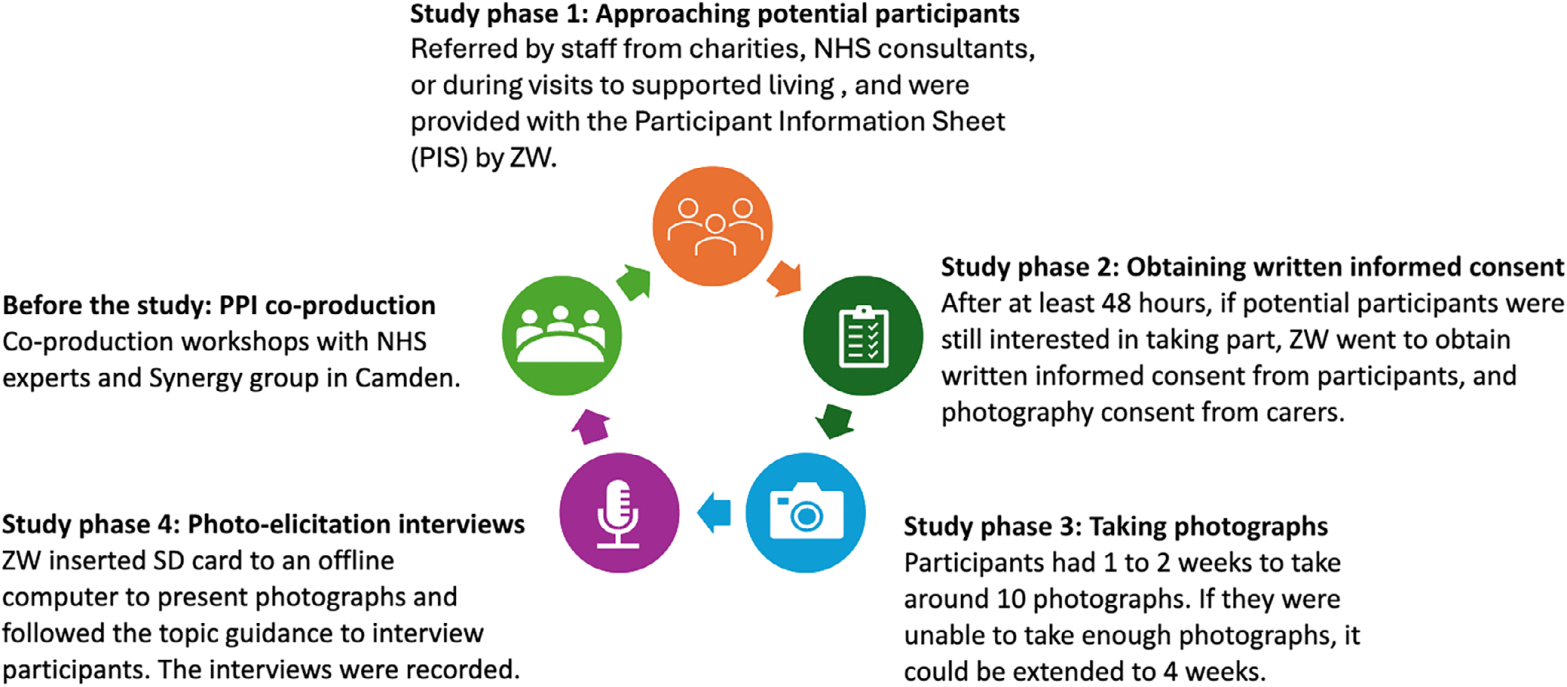
Study procedure.

The photo‐elicitation process was designed to foster mutual respect rather than reinforce hierarchical researcher–participant dynamics. It gives participants more control over the narrative, values their unique perspectives as central and changes the dynamic from a researcher‐led interrogation to a more collaborative, respectful dialogue centred on shared photographs. Figure 1 demonstrates the procedure of this study step by step. After at least 2 weeks, Z.W. arranged an in‐person meeting to carry out the photo‐elicitation interviews which were audio‐recorded. We used a semi‐structured interview guide (Appendix A) to explore what types of social participation they were engaged in and what factors supported or hindered this. In the case of the few participants who did not take any photographs, or who said that there was nothing in their life that could be photographed, the researcher (Z.W.) interviewed them without photographs but used their timetable as anchor points for discussion. Z.W. showed the participant all photographs they had taken and asked them to describe their social participation based on the photographic content and talk about how these photographs reflected their social participation challenges and barriers. Z.W. also prompted them to describe any social participation they desired. Following the interviews, the researcher gave participants an easy‐read feedback (Appendix B), asking them to rate how they felt when taking part in this project. It consists of the questions with possible answers ‘I was very happy with it’, ‘I was quite happy with it’, ‘I was quite unhappy with it’, ‘I am very unhappy with it’ and opening questions. To ensure impartiality, participants were asked to complete the feedback on their own. However, they could ask the researcher (Z.W.) or carers for clarification if they had any questions. Participants received a £20 shopping voucher as an acknowledgement of their time in taking part.

### 2.7 Patient and Public Involvement Around Engagement

The researcher conducted a Patient and Public Involvement (PPI) workshop with a group of six self‐advocates with mild intellectual disability to discuss the questions of methods, recruitment and facilitating people with intellectual disability's participation in the study. We gathered their feedback on the easy‐read version of the PIS and consent form to make them more accessible and understandable. In the meeting, the group reviewed the PIS and consent form in detail, providing feedback on clarity and suggesting improvements to make the content more understandable. This process continued until the group members were satisfied with the wording. Additionally, the group offered insights on whether the project was meaningful for people with intellectual disability and if the actions proposed in the project were appropriate and beneficial to their lives.

### 2.8 Analysis

We transcribed (using Happyscribe.com) and anonymised interviews, segmenting raw textual data into meaningful categories using MAXQDA 2020 to organise the data. The research team met eight times between December 2023 and August 2024 to discuss, develop, and finalise themes, using a reflexive thematic analytic approach, identifying similarities and differences in the data and resolving discrepancies, organising categorised data into preliminary codes. We discussed preliminary themes and deductively coded transcripts, following Braun and Clarke's reflexive thematic analysis guidance (Braun and Clarke 2021). Z.W. then considered all the data, consulting the wider research team throughout and integrating data into the final analysis. We included diverse and different experiences and voices, rather than emphasising the frequency of themes. We presented key themes and quotes, referring to illustrative participant photographs. We used the photographs as prompts to help participants recall their social participation and to facilitate the research team understanding of participants' experience.

## 3 Results

### 3.1 Participants

The participants' ages ranged from 50 to 95 years (mean 63.9, SD = 11.5). There were five females (36%) and eight males (57%). One participant preferred not to disclose their gender. Ten (71%) participants had mild and four (29%) moderate intellectual disability. Thirteen (93%) participants reported being single and one (7%) was divorced. All of them spoke English and lived in supported living. The characteristics of the sample are shown in Table 1.

**TABLE 1:** Characteristics of participants taking part in this study.

Project ID	Age	Gender	Marital status	Self‐identified ethnicity	Severity of intellectual disability	Employment	Self‐reported mental health diagnoses
1	52	Male	Single	White British	Mild	Unemployed	None
2	60	Male	Single	White British	Mild	Unemployed	Recurrent depression
3	60	Female	Divorced	Mixed: Black Caribbean/White British	Mild	Voluntary jobs (part‐time)	Schizoaffective disorder
4	63	Female	Single	Mixed: White other/Asian	Moderate	Unemployed	None
5	68	Male	Single	Mixed Black Caribbean/White other	Mild	Unemployed	Bipolar disorder
6	57	Male	Single	White British	Mild	Voluntary jobs (part‐time)	Depression
7	61	Female	Single	White British	Mild	Unemployed	None
8	55	Male	Single	White British	Mild	Unemployed	Dissociative disorder
9	77	Male	Single	White British	Mild	Unemployed	None
10	71	Male	Single	White British	Moderate	Unemployed	Bipolar disorder
11	95	Female	Single	White British	Moderate	Unemployed	None
12	65	Prefer not to disclose	Single	White British	Moderate	Unemployed	Anxiety
13	60	Male	Single	White British	Mild	Paid job (part‐time)	Prefer not to disclose
14	50	Female	Single	White British	Mild	Paid job (part‐time)	Depression and anxiety

The median number of photographs taken by participants was 19, taken over an average of 17 days, using either participants' smartphones, or project digital cameras (see Table A1 in Appendix C). Three participants did not take any photographs. Interviews lasted between 18 and 105 minutes. Participants were offered extra in‐person or telephone interviews if they did not complete the interview in one sitting. Thirteen (93%) participants completed the feedback after the interview, and all responded that they were ‘very happy’ or ‘happy’ with the interviews.

### 3.2 Social Participation and Support Network

**FIGURE 2:**
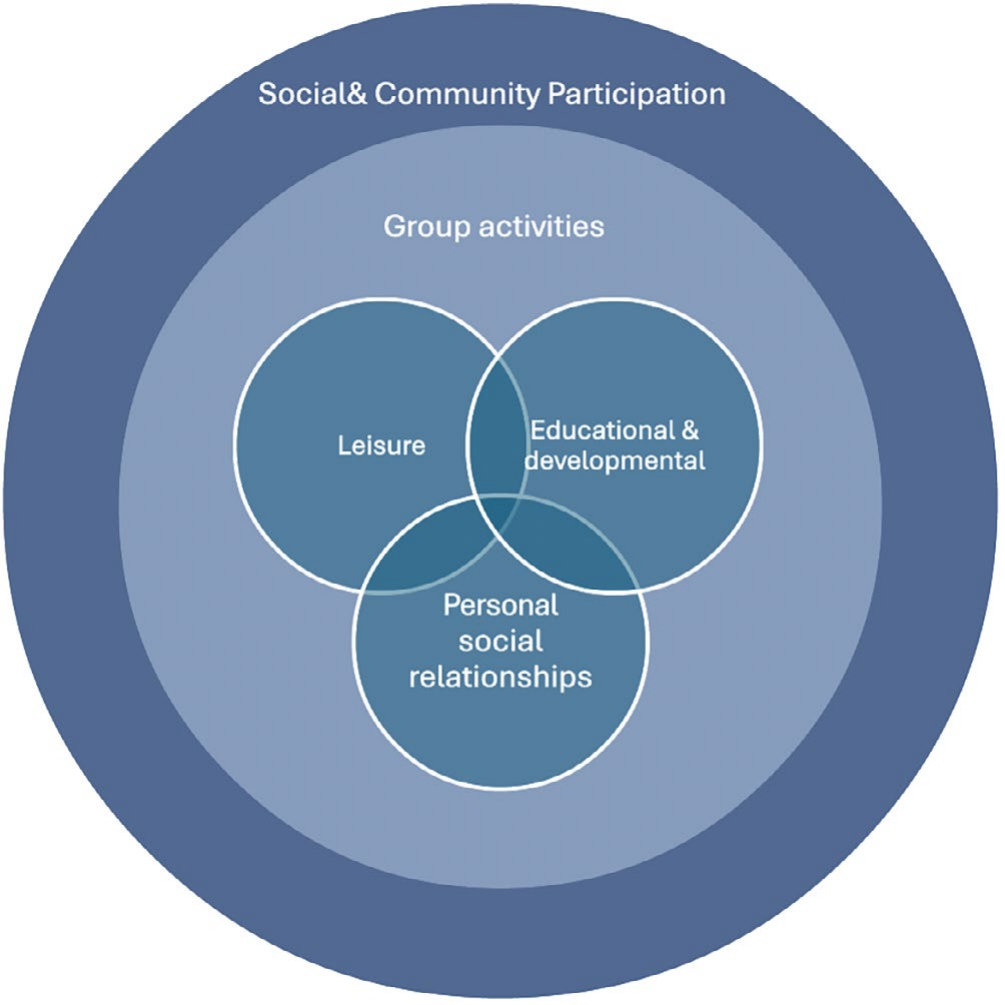
Type of social participation and activities described by people with intellectual disability in study interviews.

**FIGURE 3:**
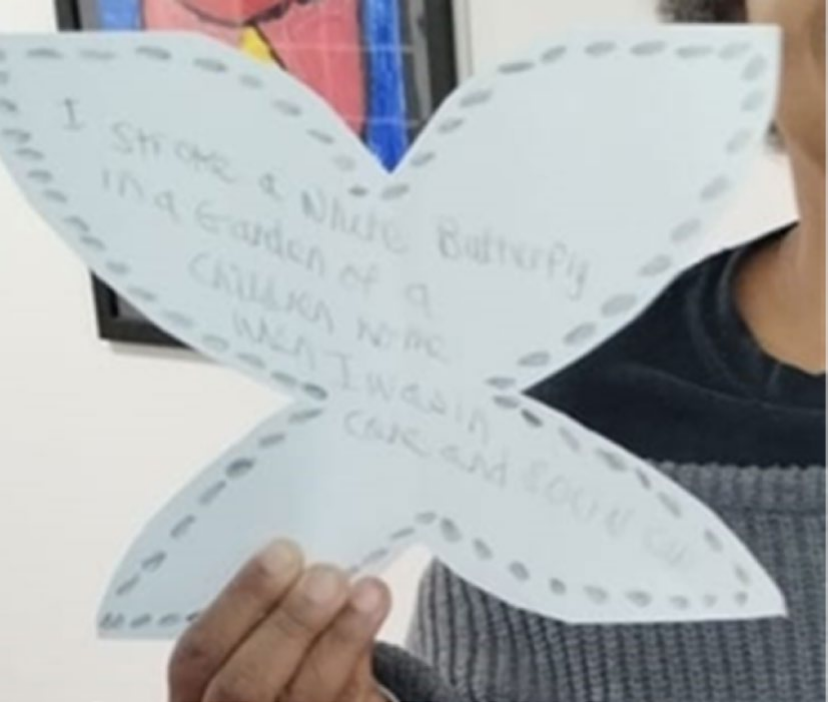
Black and white butterfly: ‘It's a black and white butterfly … And then a lady did a butterfly for me. A big one … and a small one … I can't remember what I wrote on it. Oh, yeah, I remember that. It's what I did in the past. When I was in care in a children's room, a white butterfly came towards me, and I stroked it. I stroked a white butterfly’ (P03, 60 years old, female, Black Caribbean and White British).

**FIGURE 4:**
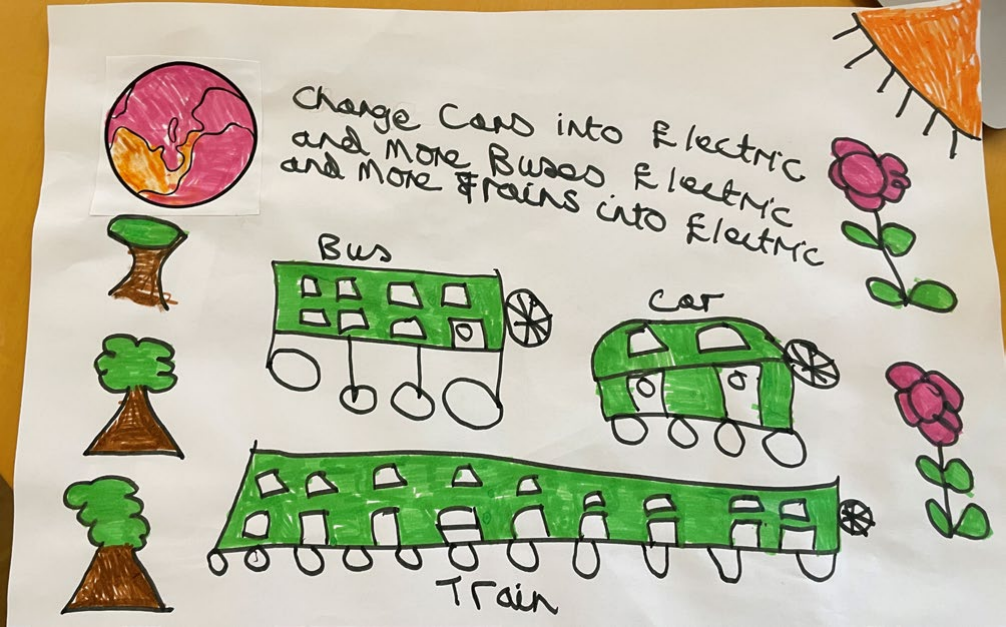
Photograph taken by study participant reflecting on their art session in a community centre: ‘We were talking about how to help the earth by pollution, having less pollution. And then change cars into electric and more buses into electric. And more trains into electric. So I draw a bus, a car, and a train…’ (P03, 60 years old, female, Black Caribbean and White British).

Participants showed photographs of multiple types of social participation which they discussed with the researcher. Figure 2 summarises the types of social participation people engaged in. Personal social relationships were a fundamental part and central to all participants' lives; these relationships were sustained through everyday activities such as going out with carers, socialising with housemates in the supported living and contacting families and friends. Many participants in the study were able to engage in group leisure activities such as group museum visits, walking challenges and charity art sessions; for example, P03 was excited about her artworks; see Figures 3 and 4. Fewer people were able to attend educational and developmental activities, such as going to adult college or other day courses. A small number had paid/voluntary jobs, which provided additional opportunities for social participation.

**FIGURE 5:**
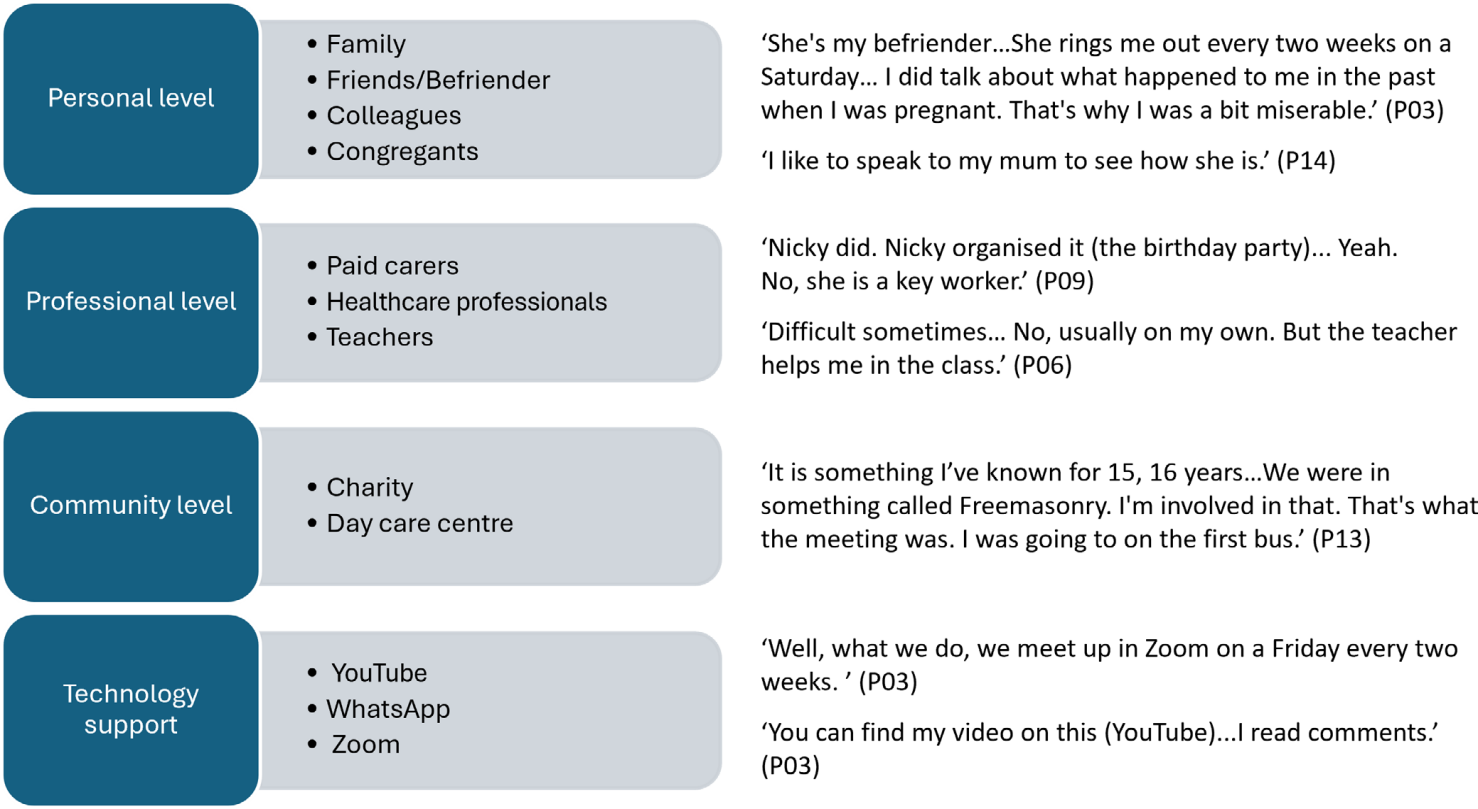
Networks of support received by people with intellectual disability.

As shown in Figure 5, participants described sources of support and information about social participation, which we labelled as support network. Everybody received help from support workers, but some received additional support from families and friends, colleagues and fellow congregants from religious institutions (if any), healthcare professionals, teachers, charity and day care centres and few of them used online applications (e.g., WhatsApp) to contact families and friends.

### 3.3 The Photo‐Elicitation Themes

In the photo‐elicitation interviews, participants reflected on their wish for social connection and the various challenges depicted in the photographs they captured while socialising or attempting to engage socially with others. Themes and subthemes are described below and in Table 2.

**TABLE 2:** Themes and subthemes.

Themes	Subthemes
Negotiating belonging in a world of barriers	Sense of belonging and connectedness Fulfilling emotional needs Doing new things
Obstacles go beyond intellectual disability	Psychological and physical barriers Practical challenges Social stigma Loss of friends Need encouragement and company
Support must be tailored and personalised	Information and planning Social skills development Need organisers and facilitators

### 3.4 Theme 1: Negotiating Belonging in a World of Barriers

#### 3.4.1 Sense of Belonging and Connectedness

Being connected with family and close persons helped participants to gain a sense of belonging and some reported that they would like to have more close people. Some participants talked about their partner relationships and two participants expressed their desires for intimate relationships during the interviews:

Because I want to find a girlfriend to cheer me up or go out with her or go for walks. If she can walk far as I can. (P02, 60 years old, male)

I'd like to make a person to be my girlfriend … And if I did, I would treat her and spend the money on her… when the staff go out with me and take me out, I pay for the drinks and the cake and food out for my money with the kindness of my heart. (P10, 71 years old, male)

#### 3.4.2 Fulfilling Emotional Needs

Participants often found emotional fulfilment and built up confidence through social participation. P06 believed seeing people gave him more opportunities to develop confidence, and P13 and P14 believed social participation made them feel needed, valued, and happy.

(I can) Get my confidence … talking with them … I try to see other people. (P06, 57 years old)

Yeah, or feel valued … With around other people … I do feel good being with other people … I can share … They share with me sometimes. (P13, 60 years old, male)

I help her a lot on Saturday shopping … (I feel) happy … (I feel be) needed. (P14, 50 years old, female)

#### 3.4.3 Doing New Things

Participants liked trying new things; different activities made them feel excited and provided them with new information and experiences. P13 believed exploring new places and engaging in different new activities could encourage him to go out more often.

I look forward to it … Meeting people in a different environment, in a public environment rather than that. (P13, 60 years old, male)

### 3.5 Theme 2: Obstacles Go Beyond Intellectual Disability

#### 3.5.1 Psychological and Physical Barriers

Most participants reported emotional and psychological struggles, including embarrassment, frustration, dependence on carers and issues related to stigma and self‐perception. P02 said he felt embarrassed when he was participating socially, which reduced his confidence and willingness to socially interact.

And sometimes it's hard for me because of my learning difficulties. I can't read that much … Embarrassing for me … Sometimes it's frustrating when you can't do it right. I get annoyed for myself … I do get a bit nervous, but people don't realise that I am a bit shy … I had to get more confident…. (P02, 60 years old, male)

Participants reported that they had traumatic experiences such as being raped, bullied, abused, or robbed. These led to anxiety, lack of trust and feelings of insecurity in social contexts. P01 and P03 both experienced rape in the past, which left them feeling fearful of dark places and certain neighbourhoods. P03 consequently decided to leave her neighbourhood and move to a new area, resulting in the loss of her existing social networks, but she did not wish to talk about it further.

Because of my Down syndrome … I was bullied … When I was young … (They) call me names about much away. Took me to the basement … Yeah, it happened in the school itself. There was a big shower room that whipped my clothes. Took them off. One pushed me down on the floor, turned me over … I did not tell any teacher … They raped me … I don't like the dark…. (P01, 52 years old, male)

Well, I was shocked to find out that man (she saw him on the street) who raped me in 2010, is that a prison? That's what I felt shocked … It'd be nice to get security. (P03, 60 years old, female)

Some participants preferred to socialise with others who also had intellectual disability. They felt it was safer as they would not be taken advantage of. P08 expressed his worries:

Well, in my time, I've had some experiences, some scary people, and I've got away from them and dealt with me to stay away from them because I know who they are. And I keep them at arm's length because some people not say who they really are. And you can find them out. You do find them out. And they're the ones that are dangerous. They're the ones that rob you for money. (P08, 55 years old, male)

Some participants were physically unwell or had sensory impairments and needed extra help to go out. Three participants expressed that being physically unwell made it difficult for them to go out.

My leg is swollen … Yeah. A lot of tablets. I take back my leg pretty stronger … Went on hospital with my ears and my legs. (P07, 61 years old, female)

I am almost deaf. (P10, 71 years old, male)

I had to take my time walking up and down the stairs, one by one, one by one … and I can't run because I got arthritis on my knees…. (P03, 60 years old, female)

#### 3.5.2 Practical Challenges

In addition to their own health and emotions, participants also reported many practical difficulties when they were trying to socialise, including communication barriers, accessibility and mobility issues, scheduling conflicts, choices of social places and financial difficulties. Although they had time and ability to go out, they did not know when and where to go. P02 lost contact with a close friend because she went to hospital, and P06 did not know where and when he could find more social opportunities.

This lady, she's in hospital. We're close friends, I like hugging her … And I tried to phone her and this bloke, a friend of ours, I said, ‘Is she all right?’, ‘No, she's in hospital.’ I said, ‘Do you know what hospital?’ And then he hung up and that really hurt! … So, I got to wait till she comes back out of hospital because it worries me now. (P02, 60 years old, male)

I want more (social) … Have more company … (I need help to find) Good places. Wonderful places. (P06, 57 years old, male)

Also, P14 reported that the sessions held by charities that he was interested in had scheduling conflicts, preventing him from attending them.

No, it's not easy (to see friends). It's not enough of the right time … I'm looking for a fairly regularly joining here … Unfortunately, most Wednesdays, I'm over at Head Office … If I finish at 1:00, I'm not going to get in there for lunch … I like both. I like both and can't do them the same time, but anyway. (P14, 50 years old, female)

Participants reported that they preferred to visit social places which were easy for them to go to, or easy to access, for example, the community activity centre, or the breakout area in the college. These places were usually located within walking distance. P01 and P06 both found that close places made it easy to socialise.

I found it easy, because it's next door to my mom's house. (P01, 52 years old, male)

I do a breakout area … There's a breakout area where we get a lunch. That's good … It's in the college. (P06, 57 years old, male)

Some participants reported difficulties in financial management, affecting their ability to make independent purchasing decisions and go out by themselves. For example, P10's carer set a £10 limit at a time to help him manage his spending.

Well, it is a bit difficult to choose exactly what I want to buy. It takes me hours to make up my mind what I want to buy because I only had 10 pounds, but I don't know. I just like going shopping … They stopped helping me because it's difficult for me to learn how to shop because I'm not sure on money. (P10, 71 years old, male)

#### 3.5.3 Social Stigma

Social stigma here refers to negative stereotypes and prejudices about participants with intellectual disability, which led to the exclusion and marginalisation of participants. P02 expressed his experience of being teased by people in his neighbourhood.

They call me freak … Because of my learning difficulties. I've got to learn these difficulties … But people know (my disability) and they try to wind me up, trying to make me angry…. (P02, 60 years old, male)

#### 3.5.4 Loss of Friends

Some participants' social networks shrank as they aged. A few participants highlighted the challenges related to maintaining and forming social connections, and with loss of friends. P01 and P07 both lost their close friends death.

I knew her at school … I go to pub (with her) … She passed away in that room. (P01, 52 years old, male)

It felt good for the work we do. Amazing work. Christine, my teamwork just passed away. She died … She died in the hospital when I was in Dublin. (P07, 61 years old, female)

#### 3.5.5 Need Encouragement and Company

Participants found it easier to take part in social activities if someone else encouraged them to go. P08 said carer's encouragement supported him to make the decision to go out.

I'll go again when the time's right. When the time comes up again, I'll just go in … You (carer) told me about it and you … (otherwise) I would have stayed at home. So I just went in it. (P08, 55 years old, male)

Participants reported that they preferred it if carers accompanied them. This suggests that because of lack of confidence or habit, rather than a lack of ability. P12 was anxious when talking about going out without carers' support.

(Researcher: Why can't you go out by yourself?) I've never done it. I've never done it. (P12, 65 years old, gender prefer not to disclose)

### 3.6 Theme 3: Support Must Be Tailored and Personalised

Participants reported different needs and difficulties of social participation, and required tailored support, personalised to the different needs of participants.

#### 3.6.1 Information and Planning

Participants found up to date activity timetables could help them in social planning. Some charities emailed updated timetables to participants monthly, and supported living also provided timetables to residents but sometimes these were out of date. P10 complained that his timetable was not up‐to‐date and P13 found the monthly activity plan was useful for him.

No, I am not happy because they haven't made me activity list … Yeah. What you do each day…. (P10, 71 years old, male)

There's a timetable. As I said, I've been attending that facility for the best part of 20 years … I know what is happening and when … It's useful to know that before you turn it up. (P13, 60 years old, male)

#### 3.6.2 Social Skills Development and Training

Participants reported that they were not good at communicating and they needed social skills training, for example, about learning to listen to others, or reading facial expressions. P13 expressed his difficulties in reading the social signals of others.

It's a lot of things did make it difficult … Reading signals. I don't know what the other think. But some feels … Reading other people's feelings (is difficult). (P13, 60 years old, male)

**FIGURE 6:**
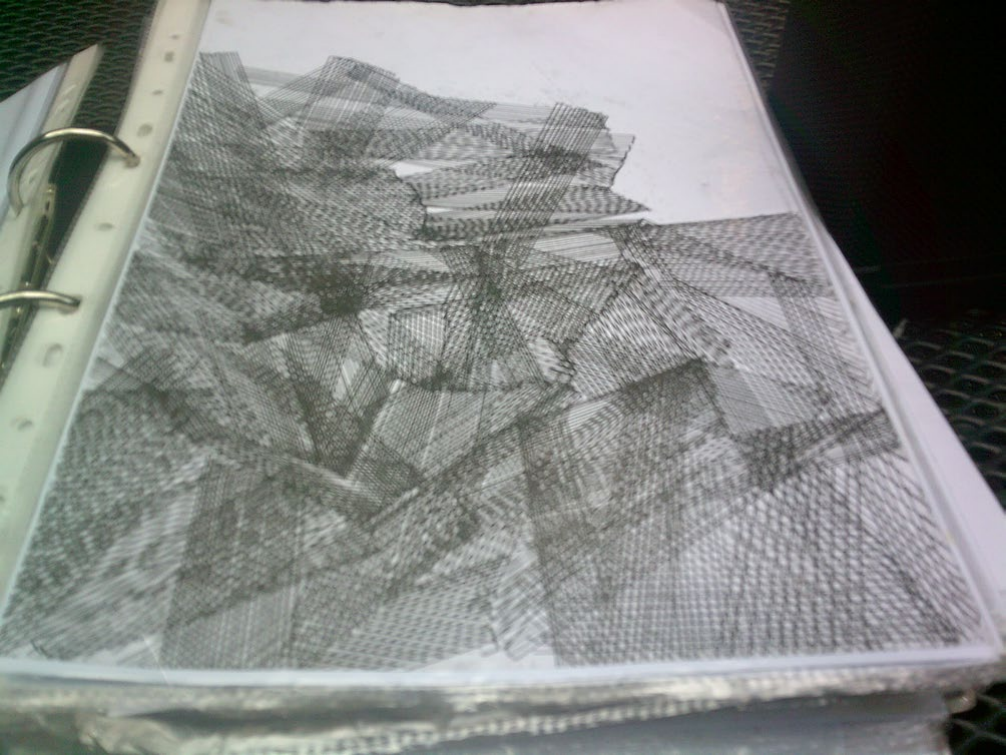
Angry management: ‘I do drawing … So I let the anger come out on the paper instead of myself and everybody else. And then some people look at it and go, “where have you been? What are you doing?” I said, “No, it's just the way I do it.”’ (P02, 60 years old, male, White British).

Some participants reported that they found it difficult to control their temper, but were trying to use techniques to ensure they did not show inappropriate frustration or anger. P02 shared his experience of managing anger by drawing (see Figure 6).

Have you noticed I'm always doing this to my hand? That's a pressure point … I do a pressure point on myself … No, stop trying not to get myself frustrated or angry. (P02, 60 years old, male)

#### 3.6.3 Need Organisers and Facilitators

Usually, paid carers organised social activities and provided social places and resources to support participants. P07 mentioned that her carer was really helpful by keeping her informed about social activities and sharing information with her.

Give all my information (about walking challenges). Give my paper … She let me know. (P07, 61 years old, female)

Participants reported that they need someone whom they trust to break the ice in a social situation to build up the initial connections. P06 shared how challenging he finds it to initiate conversations in social settings, while P08 felt that he could only comfortably interact with others if introduced by someone he already trusted.

Don't want to think what to say to them (at first). (P06, 57 years old, male)

And it's my choice (to be friend or not) … I just want to keep myself to myself … They're not like you or carer … (Carer: so, you trust them if they are introduced by someone else?) … That's right. That's it. That's the key. (P08, 55 years old, male)

## 4 Discussion

### 4.1 Main Findings

In this qualitative study using photo elicitation methods, we found that participants with mild/moderate intellectual disability engaged in multiple types of social participation organised or facilitated by support workers from supported living, charities and organisations and local community as well as exploring activities themselves. They received support from carers, family and friends, professionals, community and additional technology assistance to enhance and maintain their social participation. However, our findings also indicated that participants wanted more social connections but encountered barriers, many of which extended beyond intellectual disability to physical illness or experiences that led to them feeling unsafe. This highlighted the need for tailored support to help them with these challenges and increase their participation in social activities.

#### 4.1.1 Negotiating Social Belonging in a World of Barriers

Participants felt that they gained a sense of belonging and connectedness from social participation, and they would like to have more opportunities to participate. This desire for belonging and connection may motivate them to attend social events and participate in the community (Van Asselt et al. 2015). A sense of belonging, however, goes beyond mere social inclusion; it involves feeling attached and connected within specific spaces and groups (Hall 2010). Participants also wanted to fulfil emotional needs through social participation. They reported that they experienced emotional fulfilment through social participation, particularly when helping other people. This is similar to a study which found that friendships could give people with intellectual disability a sense of being personally valued (Mason et al. 2013). Participants also reported a desire to do new things and wanted to explore environments beyond their usual settings.

Although the wish for belonging, emotional fulfilment and novelty drove participants to have more social connections, the way in which they built up and maintained their social participation was shaped by their past experiences and preferences. Previous studies explored the social preferences of people with intellectual disability. One study found that people with intellectual disability tended to spend time with people who provided support to them, shared similarities and were perceived to be trustworthy (Fulford and Cobigo 2018). Within the social networks of people with intellectual disability living in communities, a quarter of all network members were other service users with intellectual disability and a further 43% were staff and around a half of social network members of people with intellectual disability were said to have provided company and to ‘keep an eye on’ or ‘look out for’ them, providing invisible support (Forrester‐Jones et al. 2006). There is also a preference for reciprocal relationships in which they feel they are treated equally (Callus 2017). Our findings suggest that people with intellectual disability preferred to become friends with others who also had intellectual disability, citing mistrust towards those without intellectual disability due to past experiences of victimisation. When building relationships with those without intellectual disability, they are more likely to accept them if introduced by organisers or facilitators they trust, such as paid carers, charity staff or friends with intellectual disability.

#### 4.1.2 Obstacles Go Beyond Intellectual Disability

Additionally, different barriers appeared to hinder their ability to socialise. For example, they often face multifaceted communication challenges, which can lead to social isolation (Ellis et al. 2023). Our participants reported difficulties related to communication, potentially linked to their intellectual disability, such as struggling to read facial expressions during conversations, difficulty breaking the ice and uncertainty about how to initiate interactions. Three participants either reported or were observed by the researcher to struggle with controlling their anger, which created tensions in friendships and made it harder to sustain social relationships. Additionally, participants expressed feelings of embarrassment and a lack of confidence due to their intellectual disability, often attempting to conceal it. This may partly reflect impairment in social cognition and consequent social skill difficulties experienced by people with intellectual disability, particularly those with autism. This indicates the potential benefit of social skills training, which is recommended by the National Institute for Health and Care Excellence for adults with autism (NICE 2021; Rose et al. 2021; Skwerer 2017).

Beyond challenges associated with intellectual disability, psychological and physical factors also exert a negative impact on social participation, manifesting in difficulties with independent mobility and decision‐making without external support. Changes in living arrangements, the loss of parents or adult siblings who provided support and companionship and age‐related health and mobility challenges can all contribute to a decline in social opportunities (Stancliffe and Hall 2023). Many people with intellectual disability relied on carers and were reluctant or unable to socialise independently. As a result, people with intellectual disability may struggle to expand their networks or make decisions, thus reinforcing the cycle of dependence on their carers and feeling they have insufficient social connections.

Despite having the time and desire to socialise, many participants were unsure of when and where to socialise, due to practical challenges, such as limited information, scheduling conflicts, accessibility and financial management skills. Poor knowledge or literacy could result in a lack of expressed demand for support. A potential solution is to facilitate older people with intellectual disability becoming volunteers or joining neighbourhood leisure clubs, which may create more opportunities for social participation by making new acquaintances and expanding social networks (Boland et al. 2025).

Victimisation and stigma were other barriers to social participation for people with intellectual disability as half of our participants reported experiencing negative or traumatic events in their lives. This may contribute to the expressed preference for socialising with people with intellectual disability. A study found that despite widespread support for social inclusion, stigma against people with intellectual disability remains persistent (Scior et al. 2020). They may have been singled out or ignored due to their disability, leading to social exclusion (Abbott and McConkey 2006). A Spanish study found that one in three people with intellectual disability have suffered bias victimisation; they became more vulnerable and tended to face a higher number of different types of bias victimisation than those who haven't been targeted by bias (Díaz‐Faes et al. 2023). Understanding stigma and normalisation can help us gain insights into what people with intellectual disability need and desire, but from which they may have been excluded (Harrison et al. 2021). In the future, we can implement interventions aimed at raising awareness and promoting understanding of intellectual disability within communities.

Loss of friends further complicated the social participation of ageing adults with intellectual disability, particularly for participants who initially had small social networks, which were further diminished; therefore, support in maintaining and facilitating contact with family and social networks is needed (Thalen et al. 2023). Moreover, they needed encouragement and company from someone whom they trusted to motivate them to go out, as they may lack the confidence or resources to initiate participation independently (Thalen et al. 2023).

#### 4.1.3 Support Must Be Tailored and Personalised

These difficulties contribute to a progressive narrowing of social participation of participants, which might create a detrimental cycle that further exacerbates their social disadvantages. Importantly, these barriers were not solely attributable to their intellectual disability, underscoring the need for tailored support. Examples are provision of transportation, social skills training, or simplified communication tools and how to use them in social interactions through role modelling. Therefore, personal‐centre planning may help people with intellectual disability by recognising and responding to personal needs and empowering them to overcome some of these challenges (McCausland et al. 2022). Those who were able to engage in more complex social activities (e.g., work) also required less help to organise social activities, for example, going out alone to visit a friend's home. In contrast, participants who were less capable required greater support from others; thus, building circles of support is meaningful for people with intellectual disability (Araten‐Bergman and Bigby 2022).

#### 4.1.4 Technology Use

Many participants were able to use online applications, such as Zoom meetings or WhatsApp, to stay in contact with their families and friends, so these were valuable and accessible tools to provide social opportunities. Our findings are consistent with another UK study and a scoping review of all the literature, which reported that people with intellectual disability had positive experiences using social media to develop friendships, social identity and self‐esteem and enjoyment, but there were safeguarding concerns and other difficulties caused by literacy and communication skills (Anderson et al. 2023; Caton and Chapman 2016). Sometimes, online identities can differ from those in the ‘real world’ and people with intellectual disability do not necessarily disclose their intellectual disability (Löfgren‐Mårtenson 2008), as they feel that this may mean they avoid stigmatisation (McClimens and Gordon 2008), so online social networking can be a partial substitute for real‐life social participation (Holmes and O'Loughlin 2014). Based on these advantages, although virtual social contact cannot replace all in‐person social contact, engaging in virtual social contact may help during times of isolation (Bakkum et al. 2022).

### 4.2 Strengths and Limitations

In this study, we engaged people with mild and moderate intellectual disability by using photo‐elicitation to hear their own narratives of and reactions to social engagement. We purposively recruited people from a variety of organisations and of a wide demographic characteristics. This allowed us to collect rich and complex information and obtain valuable insights into their lives.

There are limitations to our study. First, some participants did not take any photographs during the project either because there was no carer available to help them to take photographs, because they judged that there was nothing which was worth photographing, because they believed taking photographs in public places would get them into trouble, or they forgot. For these participants, interviews were not based on photograph content. Second, a few participants were unable to recognise the photographs they had taken and so were unable to discuss the content of them in detail. To address these issues, future studies could recruit carers to provide better support to participants. Third, we only recruited participants who were living in London and so the findings may not be generalisable to rural areas or other countries. Expanding recruitment outside London in future research could enhance broader applicability. Fourth, participants all spoke English, so findings may not be generalised to other groups. Future studies could include interpreters to facilitate participation among diverse linguistic populations. Fifth, we recruited the participants from charities who replied to our invitation, those charities may have had better services, or were more interested in making social participation better. To mitigate this limitation, we could recruit participants from more diverse sources in future research. Sixth, our PPI workshop with self‐advocates was helpful for guiding the study but people who participated in the PPI workshop were normally those with strong community commitment and others who did not take part may have different opinions. Involving people from more diverse backgrounds in future PPI workshops could enrich input. Finally, we recruited people with mental capacity to consent to participation, so the findings do not tell us about people with more severe intellectual disability.

## 5 Conclusion

This study used the photo‐elicitation method to explore social participation among community‐dwelling people with mild or moderate intellectual disability. Photo‐elicitation methods helped capture powerful imagery and enhanced participants' memory during the interviews and facilitated communication between the researcher and participants. Evidence from this study suggests that people with intellectual disability enjoy social participation and are aware of the benefits of being in the company of others sharing the same interests. However, they had multiple difficulties accessing venues and opportunities not only because of their intellectual disability, but also due to past trauma, chronic illness and a lack of support in accessing services beyond those organised for their peers. Practically, our findings suggest that people with intellectual disability could be provided with more tailored support to increase their social participation by targeting specific difficulties. Further research could use our findings for a co‐produced intervention to reduce loneliness and enhance social connectedness in people with intellectual disability.

## Author Contributions

**Zuyu Wang:** conceptualisation, design, investigation, data curation and analysis, writing – original draft preparation (lead), writing – review and editing, project administration. **Andrew Sommerlad:** conceptualisation, design, data analysis (theme development), writing – review and editing, supervision. **Joan K. Monin:** writing – review and editing, supervision (supporting). **Angela Hassiotis:** conceptualisation, design, participants recruitment, data analysis (theme development), writing – review and editing, supervision. **Gill Livingston:** conceptualisation, design, data analysis (theme development), writing – review and editing, supervision.

## Ethics Statement

This study was approved by NHS Health Research Authority: West Midlands‐South Birmingham Research Ethics Committee (23/WM/0091).

## Consent

All study participants provided written informed consent.

## Conflicts of Interest

The authors declare no conflicts of interest.

## Data Availability

The data are not publicly available as they are easily identifiable.

## Appendix A Hear Our Voice Study—Photo‐Elicitation Topic Guidance

### Participants

The photo‐elicitation interview will be one‐to‐one with people living with learning disability who can also include their family or paid carer if they wish.

### Script for Photo‐Elicitation Interview

#### Before the Photo‐Elicitation

- Insert SD card and show all photos on computer.
- Introduce myself and remind the participant why I am interviewing them.
- Provide a background to the Hear Our Voice study (see below).
- Inform the participant that the photo‐elicitation interview will not last under an hour (60 min).
- It is important to remember that the participant is the expert during the photo‐elicitation interview.

Tips: The interview should conversational and be as natural as possible to encourage the participant to provide detailed information. Please remember to respond to the participant, you can use body language or say something to encourage him/her. Italicised text below should be read to the participant and normal font text is to support the interview.

### Aims of the Hear Our Voice Study

*Thank you for attending this interview. My name is x and I am from University College London*.

*Thank you for taking photos for our study. I would now like to talk about them*.

I: *First of all, I will give you a Notecard and a piece of paper to help you organise your stories, in this card, there are 5 questions*.

Then I will read these five questions out giving time for participants to answer each question.

**Table:**

Notes
*Q1. Tell me about what is happening in this photo*.
*Q2. Did you enjoy it?*
*Q3. What makes it easy or difficult for your social situation?*
*Q4. What can we do to help?*
*Q5. Would you like in the future to do more or less of this or do it the same or differently?*

### Participants' Narratives of Their Photos

In this part, the participant's narratives of their photos (from his/her favourite ones) should include Questions 1–3. If not, I will encourage the participant to continue describing his/her photos or remind him/her of which questions he/she still need answers to if they can.

I: *I am just going to give you two minutes for you to recall what was going on in this photo on your own, I would like you just to think about it. Then we will have a brief round‐up of some of the things that you see*.

I: *Tell me, what do you see (slowly), please feel free to say what is obvious, for example, this is a tree, and if you see something else I also want to hear that too, so go ahead*!

I: *OK, can you please tell us what is happening in this photo?*

I: *That is interesting/it is good to know this*.

I: *If you have any comments please feel free to share with me!*

**(Repeat these questions for each photo, one by one and be patient.)**

Q. *Before we finish, is there anything else you would like to mention that we have not already covered?*

*Thank you for taking part today. If you have any other thoughts or would like to ask me any questions please let me know*.

Check if participant has any questions answered before leaving.

End.

## Appendix B Feedback Form

### PART 1

This question is about your feeling during the participation.

### What Did You Feel During This Interview?

Please tick (√) in box.

I was very happy with it.

I was quite happy with it.

I was quite unhappy with it.

I am very unhappy with it.

### Part 2

1. What was good about taking part?
2. What was not so good about taking part?
3. How could we have made this interview better?
4. Is there anything else you would like to tell us?

Did someone else help you fill in this form, or has someone filled it in on your behalf?

Yes  No

If yes, please tick the correct box below:

Parent

Carer/support worker

Other

Thank you for taking time to complete this.

Please return this form to me in the envelope provided.

Miss Zuyu Wang

Division of Psychiatry

University College London

Floor 6, Maple House

149 Tottenham Court Road

W1T 7NF

Email: zuyu.wang.21@ucl.ac.uk

Mobile: (+44)203108229

## Appendix C

**TABLE A1:** Number of days of taking photographs, numbers of photographs and device(s) used.

ID	Duration of taking photographs (days)	Number of photographs	Device(s)
1	15	277	Camera^a^
2	12	119	Camera^a^
3	16	18	Smartphone^a^
4	14	28	Camera^b^
5	9	0	Camera^a^
6	30	0	Smartphone^a^
7	29	19	Camera^a^
8	15	15	Camera and smartphone^a^, ^b^
9	14	0	Camera^a^
10	14	60	Camera^b^
11	24	82	Camera^b^
12	24	72	Camera^b^
13	13	17	Camera^a^
14	5	19	Smartphone^a^

^a^ Participants took photographs on their own.

^b^ Participants took photographs under the supervision of a professional carer they trusted.
